# Sensory Health and Social Well-Being

**DOI:** 10.1093/geroni/igaa057.2093

**Published:** 2020-12-16

**Authors:** Corinna Tanner, Jeremy Yorgason, Joshua Ehrlich

## Abstract

Scientific inquiry into the psychological and social issues surrounding age-related sensory impairments has focused on ways in which the conditions are a catalyst for negative outcomes. However, investigating the patterns associated with negative life events can offer guidance on ways to circumvent or mitigate negative outcomes, and even to foster and facilitate the positive outcomes of growth and thriving. This symposium will present findings from individual studies that describe how social well-being among older adults with sensory impairments can be protected, by assessing social isolation as a point of intervention to maintain cognitive function and to promote post-traumatic growth, and by understanding the unique social considerations relevant to Hispanic older adults, and to improve the physical safety of older drivers with sensory and cognitive impairment by reducing exposure-adjusted motor vehicle crash risk. Authors will present both cross sectional and longitudinal, population based data, and will explore patterns and relationships between known variables associated with sensory impairments including depression, cognitive processing, cognitive functioning, social network, social isolation, driving patterns, and posttraumatic growth. Findings from the Longitudinal Research on Aging Drivers (LongROAD) study, the National Health and Aging Trends Study (NHATS), and data from a mixed methods study, underscore the reality that the negative outcomes associated with age related sensory impairments are not necessarily imminent and that there may be multiple intervention points to optimize the social well-being of older adults with sensory impairments.

